# Crystal structure and magnetic properties of (tris­{4-[1-(2-meth­oxy­eth­yl)imidazol-2-yl]-3-aza­but-3-enyl}amine)­iron(II) bis­(hexa­fluorido­phosphate)

**DOI:** 10.1107/S2056989019001531

**Published:** 2019-02-12

**Authors:** Kateryna Znovjyak, Igor O. Fritsky, Iryna A. Golenya, Tatiana Y. Sliva, Matti Haukka

**Affiliations:** a Department of Chemistry, Taras Shevchenko National University of Kyiv, Volodymyrska Street 64, Kyiv, 01601, Ukraine; b UkrOrgSyntez Ltd, Chervonotkatska St 67, Kyiv 02094, Ukraine; c Department of Chemistry, University of Jyväskylä FIN-40014, Jyväskylä, Finland

**Keywords:** crystal structure, spin crossover, spin transition

## Abstract

In the complex cation of the title compound, [Fe(C_27_H_41_N_10_O_3_)](PF_6_)_2_, the tripodal tris­{4-[1-(2-meth­oxy­eth­yl)imidazol-2-yl]-3-aza­but-3-en­yl}amine ligand is coordinated to an Fe^II^ ion through the nitro­gen atoms of three imidazole and three imino groups. The Fe atom exhibits a distorted octa­hedral geometry. In the crystal, l and d anti­podes are arranged in layers in the *bc* plane. Weak C⋯F and C—H⋯F/O contacts exist between the ligands of the complex cation and the PF_6_^−^ anions, generating a three-dimensional network. At 120 K, the Fe^II^ ion is in a low-spin state, with an average Fe—N bond distance of 1.970 (2) Å. On heating, the Fe^II^ ion converts to the high-spin state, as demonstrated by magnetic susceptibility measurements.

## Abstract 2

The title compound, [Fe(C_27_H_41_N_10_O_3_)](PF_6_)_2_, is an example of an iron(II) spin-crossover compound. In this compound, C⋯F and CH⋯F/O contacts, present between the cations and anions, extend the structure into a three-dimensional supra­molecular network.

## Chemical context   

One of the most investigated groups of switchable mol­ecular materials are the pseudo-octa­hedral Fe^II^ spin-crossover (SCO) complexes, which can change between high-spin (HS, *t*_2g_^4^ *e*_g_^2^) and low-spin (LS, *t*_2g_^6^ *e*_g_^0^) electronic states on application of physicochemical stimuli. The LS-to-HS conversion involves an electron transfer between the *e*_g_ and *t*_2g_ orbitals and is strongly coupled to structural changes in the coordination sphere of the Fe^II^ ions, affecting the Fe–ligand bond lengths and angles (Gütlich & Goodwin, 2004 ▸). The spin-state change is reversible and can be controlled, for example by the action of temperature, pressure or light. It is accompanied by a change in a number of physical properties, including magnetic susceptibility, colour, dielectric constant and NLO properties (König, 1991 ▸; Nakamoto *et al.*, 2005 ▸; Bonhommeau *et al.*, 2006 ▸, 2012 ▸). Tripod-based iron(II) complexes represent one of the well-studied classes of SCO complexes owing to the suitable ligand-field strength and readily achievable functionalization of their complex ligands (Hardie *et al.*, 2004 ▸; Seredyuk *et al.*, 2007 ▸; Klug *et al.*, 2012 ▸; Hagiwara *et al.*, 2014 ▸), particularly with aliphatic chains (Seredyuk *et al.*, 2008*a* ▸,*b* ▸, 2013 ▸, 2014 ▸).
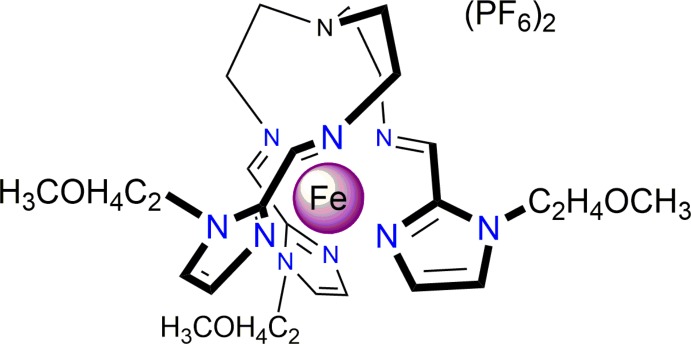


**Figure 1:**
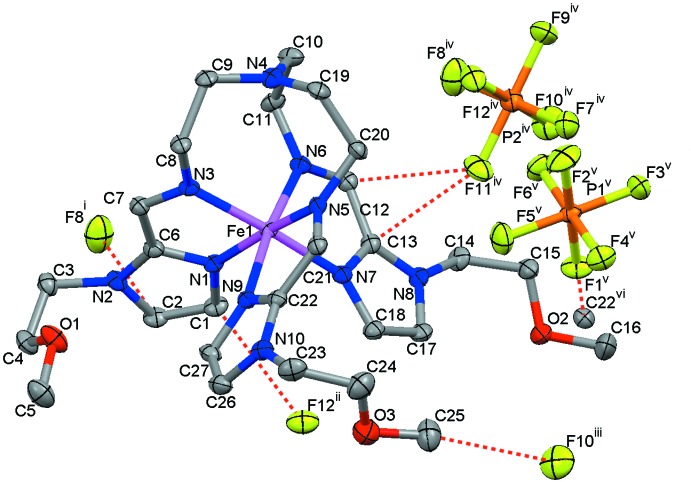
Mol­ecular structure of the complex cation and anions of the title compound showing the atom labelling. Short C⋯F contacts less than the sum of the van der Waals radii are shown as dashed lines. Displacement ellipsoids are drawn at the 50% probability level. Symmetry codes: (i) *x*, 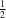 − *y*, −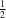 + *z*; (ii) −1 + *x*, 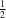 − *y*, −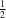 + *z*; (iii) *x*, 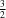 − *y*, −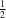 + *z*; (iv) −*x*, −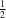 + *y*, 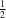 − *z*; (v) −*x*, 1 − *y*, −*z*; (vi) −1 + *x*, 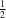 − *y*, −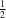 + *z*.

In this work, we report the synthesis, structure and magnetic properties of a new Fe^II^ complex based on the tripodal ligand tris­{4-[1-(2-meth­oxy­eth­yl)imidazol-2-yl]-3-aza­but-3-en­yl}amine, which can be crystallized in the presence of hexa­fluorido­phosphate anions (Fig. 1 ▸).

## Structural commentary   

The unit cell of the title compound contains two pairs of crystallographically identical complex cations of l and d chirality and eight PF_6_^−^ counter-ions (on two crystallographically distinct sites) to balance the charge. In the complex cation, the Fe^II^ ion is wrapped by three 1-(2-meth­oxy­eth­yl)-imidazol-2-yl­imino moieties, defining a pseudo-octa­hedral [FeN_6_] coordination environment (Fig. 1 ▸). The average Fe—N bond length is 1.970 Å and is typical for the low-spin state of the Fe^II^ ion (Gütlich & Goodwin, 2004 ▸) (Table 1 ▸). The average trigonal distortion parameters, *Φ =* Σ_i_^24^(60 − *θ*_i_)/24 [where *θ*_i_ is the angle generated by superposition of two opposite faces of an octa­hedron (Chang *et al.*, 1990 ▸)] and *Σ* = Σ_i_^12^(|*θ*_i_ − 90|) [where *θ*_i_ is the deviation from 90° of the *cis-*N—Fe—N angles in the coordination sphere (Drew *et al.*, 1995 ▸)] are 57.72 and 5.23°, respectively. These values are comparable to those reported previously for a similar low-spin compound with *n*-butyl substituents (Sered­yuk *et al.*, 2013 ▸). The capping tertiary nitro­gen atom, N4, is situated at a distance of 3.375 (2) Å from the Fe atom and does not participate in coordination to the metal ion. Each of the methyl­ene groups of the 2-meth­oxy­ethyl substituents directly attached to the imidazole moieties shows a *gauche* conformation, whilst the remaining methyl­ene groups are in a *trans* conformation.

## Supra­molecular features   

**Figure 2:**
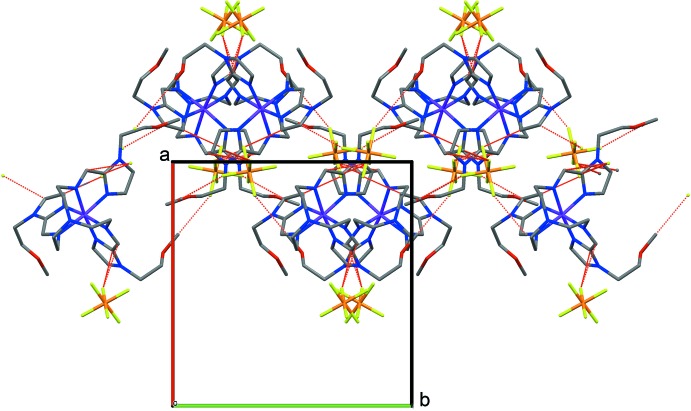
Crystal packing of the title compound viewed along [001] with C⋯F contacts shown as dashed red lines.

Supra­molecular inter­actions occur between the complex cations and PF_6_^−^ anions, with van der Waals contacts, C⋯F, lying in the range 2.934 (2)–3.137 (2) Å, linking the ions into two-dimensional layers running parallel to [011] (Fig. 2 ▸). These contacts are observed mostly for the carbon atoms belonging to the imidazole moieties of the ligand (Table 2 ▸). In addition, there are numerous C—H⋯F and C—H⋯O contacts between the complex cations and anions, extending the crystal structure into a three-dimensional supra­molecular network.

## Magnetic properties   

**Figure 3:**
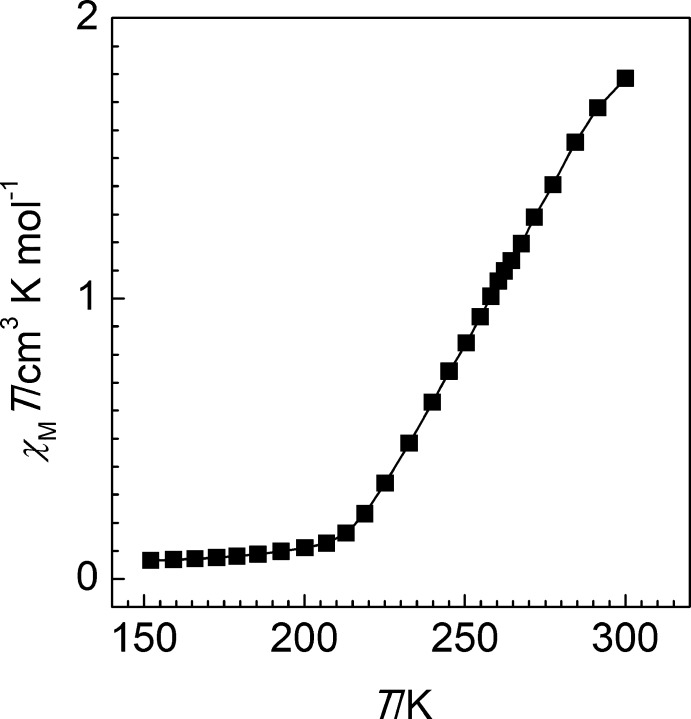
A *χ*_M_ *T versus T* plot for the title compound.

Variable-temperature magnetic susceptibility measurements were performed on single crystals (20 mg) of the title compound using a Quantum Design MPMS2 superconducting quantum inter­ference device (SQUID) susceptometer operating at 1 T in the temperature range 2–300 K. Experimental susceptibilities were corrected for the diamagnetism of the holder (gelatine capsule) and of the constituent atoms by the application of Pascal’s constants. The magnetic behaviour of the compound recorded at 1 K min^−1^ between 150 and 300 K, is shown in Fig. 3 ▸ in the form of *χ*_M_ *T* *vs* *T* (*χ*_M_ is the molar magnetic susceptibility and *T* is the temperature). At 300 K, the *χ*_M_ *T* value is close to 1.8 cm^3^ K mol^−1^, displaying at this temperature an incomplete transition of the Fe^II^ ion to the paramagnetic high-spin state (*S* = 2). On cooling, a gradual decrease of *χ*_M_ *T* value down to 0.07 cm^3^ K mol^−1^ is observed corresponding to an almost compete transformation to the diamagnetic low-spin state (*S* = 0). This corroborates well with the observed short average Fe—N bond length at 120 K and identifies the low-spin state of the central iron(II) ion.

## Database survey   

A search of the Cambridge Structural Database (CSD, Version 5.39, update November 2017; Groom *et al.*, 2016 ▸) for complexes containing the Fe^II^ ion wrapped by a tripodal ligand with a tris­{imidazol-2-yl-3-aza­but-3-en­yl}amine fragment yielded 29 hits, for which the Fe—N bond lengths lie in the ranges 1.926–2.016 and 2.151–2.286 Å for the low-spin and high-spin spin states of the Fe^II^ ion, respectively.

## Synthesis and crystallization   

A filtered solution of FeCl_2_·4H_2_O (0.043 g, 0.21 mmol) in absolute ethanol (5 mL) was added dropwise to a boiling solution of 1-(2-meth­oxy­eth­yl)imidazole-2-carbaldehyde (0.10 g, 0.65 mmol), tris­(2-ethano­lamine)­amine (0.031 g, 0.21 mmol) and [NBu_4_]PF_6_ (0.17 g, 0.43 mmol) in 5 ml of absolute ethanol. The resulting dark red–purple solution was stirred for 5 min. After standing for several days under ambient conditions, well-shaped red needles of the title compound were formed. Elemental analysis for C_27_H_41_F_12_FeN_10_O_3_P_2_ (found): C, 36.58, H, 4.98, N, 15.55%; (calculated): C, 36.77, H, 4.85, N, 15.32.

## Refinement   

Crystal data, data collection and structure refinement details are summarized in Table 3 ▸. Hydrogen atoms were positioned geometrically and constrained to ride on their parent atoms, with C—H = 0.95–0.99 Å and *U*_iso_(H) = 1.2–1.5*U*_eq_(parent atom). The highest peak is located 1.21 Å from atom C24 and the deepest hole is located 0.65 Å from atom P2.

## Supplementary Material

Crystal structure: contains datablock(s) I. DOI: 10.1107/S2056989019001531/cq2029sup1.cif

Structure factors: contains datablock(s) I. DOI: 10.1107/S2056989019001531/cq2029Isup2.hkl

Supporting information file. DOI: 10.1107/S2056989019001531/cq2029Isup3.cdx

CCDC reference: 1893862

Additional supporting information:  crystallographic information; 3D view; checkCIF report

## Figures and Tables

**Table 1:** Selected bond lengths (Å)

Fe1—N1	1.954 (2)	Fe1—N6	1.975 (2)
Fe1—N9	1.959 (2)	Fe1—N5	1.978 (2)
Fe1—N7	1.967 (2)	Fe1—N3	1.989 (2)

**Table 2:** Table of contacts (Å) shorter than the sum of the van der Waals radii

Contact	Length	Symmetry operation on atom 2
C1⋯F12	3.137 (3)	−*x*, 1 − *y*, −*z*
C2⋯F8	2.957 (4)	*x*, 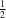 − *y*, − 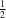 + *z*
C12⋯F11	3.044 (3)	−*x*, − 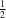 + *y*, 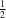 − *z*
C13⋯F11	2.934 (3)	−*x*, − 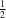 + *y*, 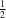 − *z*
C21⋯F1	2.902 (3)	1 − *x*, − 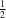 + *y*, 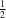 − *z*
C22⋯F1	3.022 (3)	1 − *x*, − 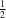 + *y*, 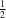 − *z*
C25⋯F10	3.074 (4)	*x*, 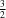 − *y*, − 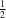 + *z*

**Table 3:** Experimental details

Crystal data	
Chemical formula	[Fe(C_27_H_41_N_10_O_3_)](PF_6_)_2_
*M*_r_	899.49
Crystal system, space group	Monoclinic, *P*2_1_/*c*
Temperature (K)	120
*a*, *b*, *c* (Å)	15.82801 (19), 14.36708 (15), 17.4210 (2)
β (°)	112.0778 (13)
*V* (Å^3^)	3671.09 (8)
*Z*	4
Radiation type	Cu *K*α
μ (mm^−1^)	5.10
Crystal size (mm)	0.39 × 0.04 × 0.02

Data collection	
Diffractometer	Agilent SuperNova, Dual, Cu at zero, Atlas
Absorption correction	Multi-scan (*CrysAlis PRO*; Agilent, 2013 ▸)
*T*_min_, *T*_max_	0.565, 1.000
No. of measured, independent and observed [*I* > 2σ(*I*)] reflections	58436, 7717, 6787
*R*_int_	0.071
(sin θ/λ)_max_ (Å^−1^)	0.631

Refinement	
*R*[*F* ^2^ > 2σ(*F* ^2^)], *wR*(*F* ^2^), *S*	0.048, 0.126, 1.02
No. of reflections	7717
No. of parameters	499
H-atom treatment	H-atom parameters constrained
Δρ_max_, Δρ_min_ (e Å^−3^)	1.43, −0.63

Computer programs: *CrysAlis PRO* (Agilent, 2013 ▸), *SHELXT* (Sheldrick, 2015*a* ▸), *SHELXL2018* (Sheldrick, 2015*b* ▸) and *Mercury* (Macrae *et al.*, 2006 ▸).

## supplementary crystallographic information

### Crystal data

**Table:**

[Fe(C_27_H_41_N_10_O_3_)](PF_6_)_2_	*F*(000) = 1844
*M**_r_* = 899.49	*D*_x_ = 1.627 Mg m^−^^3^
Monoclinic, *P*2_1_/*c*	Cu *K*α radiation, λ = 1.54184 Å
*a* = 15.82801 (19) Å	Cell parameters from 25415 reflections
*b* = 14.36708 (15) Å	θ = 4.1–76.3°
*c* = 17.4210 (2) Å	µ = 5.10 mm^−^^1^
β = 112.0778 (13)°	*T* = 120 K
*V* = 3671.09 (8) Å^3^	Needle, red
*Z* = 4	0.39 × 0.04 × 0.02 mm

### Data collection

**Table:**

Agilent SuperNova, Dual, Cu at zero, Atlas diffractometer	7717 independent reflections
Radiation source: micro-source	6787 reflections with *I* > 2σ(*I*)
Mirror monochromator	*R*_int_ = 0.071
Detector resolution: 10.3953 pixels mm^-1^	θ_max_ = 76.6°, θ_min_ = 3.0°
φ scans and ω scans with κ offset	*h* = −19→19
Absorption correction: multi-scan (CrysAlis PRO; Agilent, 2013)	*k* = −15→18
*T*_min_ = 0.565, *T*_max_ = 1.000	*l* = −21→21
58436 measured reflections	

### Refinement

**Table:**

Refinement on *F*^2^	0 restraints
Least-squares matrix: full	Hydrogen site location: inferred from neighbouring sites
*R*[*F*^2^ > 2σ(*F*^2^)] = 0.048	H-atom parameters constrained
*wR*(*F*^2^) = 0.126	*w* = 1/[σ^2^(*F*_o_^2^) + (0.0673*P*)^2^ + 4.9233*P*] where *P* = (*F*_o_^2^ + 2*F*_c_^2^)/3
*S* = 1.02	(Δ/σ)_max_ = 0.001
7717 reflections	Δρ_max_ = 1.43 e Å^−^^3^
499 parameters	Δρ_min_ = −0.62 e Å^−^^3^

### Special details

**Table:**

Geometry. All esds (except the esd in the dihedral angle between two l.s. planes) are estimated using the full covariance matrix. The cell esds are taken into account individually in the estimation of esds in distances, angles and torsion angles; correlations between esds in cell parameters are only used when they are defined by crystal symmetry. An approximate (isotropic) treatment of cell esds is used for estimating esds involving l.s. planes.

### Fractional atomic coordinates and isotropic or equivalent isotropic displacement parameters (Å^2^)

**Table:**

	*x*	*y*	*z*	*U*_iso_*/*U*_eq_	
Fe1	0.21730 (2)	0.13001 (3)	0.05119 (2)	0.01414 (10)	
O1	0.39620 (14)	−0.06399 (16)	−0.08365 (13)	0.0315 (5)	
O2	−0.14217 (15)	0.43315 (15)	−0.14865 (12)	0.0292 (4)	
O3	0.37325 (18)	0.45880 (18)	−0.02627 (15)	0.0404 (5)	
N1	0.18253 (14)	0.04448 (15)	−0.04284 (13)	0.0176 (4)	
N2	0.21644 (14)	−0.08197 (16)	−0.09714 (13)	0.0193 (4)	
N3	0.29591 (13)	0.02238 (15)	0.10600 (12)	0.0160 (4)	
N4	0.24016 (15)	0.07020 (16)	0.24537 (13)	0.0211 (4)	
N5	0.27060 (14)	0.22006 (15)	0.14262 (12)	0.0171 (4)	
N6	0.10842 (14)	0.09446 (15)	0.07388 (13)	0.0179 (4)	
N7	0.13194 (14)	0.22565 (15)	−0.01479 (13)	0.0176 (4)	
N8	−0.00982 (14)	0.27774 (16)	−0.06646 (13)	0.0198 (4)	
N9	0.31665 (14)	0.17875 (15)	0.02162 (13)	0.0175 (4)	
N10	0.42701 (15)	0.28182 (16)	0.04425 (14)	0.0208 (4)	
C1	0.13063 (17)	0.04344 (19)	−0.12578 (15)	0.0212 (5)	
H1	0.0872	0.0893	−0.1549	0.025*	
C2	0.15162 (18)	−0.0345 (2)	−0.15976 (15)	0.0220 (5)	
H2	0.1259	−0.0524	−0.2164	0.026*	
C3	0.26895 (18)	−0.16235 (19)	−0.10707 (17)	0.0233 (5)	
H3A	0.2273	−0.2075	−0.1460	0.028*	
H3B	0.2989	−0.1937	−0.0530	0.028*	
C4	0.34030 (19)	−0.1311 (2)	−0.13980 (17)	0.0258 (6)	
H4A	0.3775	−0.1848	−0.1439	0.031*	
H4B	0.3107	−0.1034	−0.1956	0.031*	
C5	0.4613 (2)	−0.0253 (2)	−0.1123 (2)	0.0354 (7)	
H5A	0.4299	0.0035	−0.1667	0.053*	
H5B	0.5018	−0.0746	−0.1172	0.053*	
H5C	0.4973	0.0219	−0.0730	0.053*	
C6	0.23381 (16)	−0.03225 (18)	−0.02678 (15)	0.0174 (5)	
C7	0.29764 (16)	−0.04447 (18)	0.05679 (15)	0.0178 (5)	
H7	0.3373	−0.0966	0.0742	0.021*	
C8	0.35865 (17)	0.01820 (19)	0.19243 (15)	0.0192 (5)	
H8A	0.3928	0.0775	0.2074	0.023*	
H8B	0.4032	−0.0325	0.1991	0.023*	
C9	0.30844 (18)	0.00118 (19)	0.25133 (15)	0.0208 (5)	
H9A	0.2789	−0.0607	0.2392	0.025*	
H9B	0.3537	−0.0001	0.3090	0.025*	
C10	0.14596 (18)	0.0435 (2)	0.21910 (16)	0.0235 (5)	
H10A	0.1111	0.0956	0.2301	0.028*	
H10B	0.1411	−0.0103	0.2527	0.028*	
C11	0.10253 (17)	0.01741 (19)	0.12726 (16)	0.0220 (5)	
H11A	0.1339	−0.0379	0.1167	0.026*	
H11B	0.0377	0.0010	0.1133	0.026*	
C12	0.03435 (17)	0.13940 (18)	0.03302 (15)	0.0185 (5)	
H12	−0.0230	0.1251	0.0358	0.022*	
C13	0.04749 (17)	0.21344 (18)	−0.01724 (15)	0.0186 (5)	
C14	−0.10876 (17)	0.2835 (2)	−0.08629 (17)	0.0237 (5)	
H14A	−0.1247	0.2420	−0.0484	0.028*	
H14B	−0.1415	0.2607	−0.1435	0.028*	
C15	−0.14067 (18)	0.3809 (2)	−0.07905 (17)	0.0240 (5)	
H15A	−0.2024	0.3789	−0.0773	0.029*	
H15B	−0.0988	0.4103	−0.0274	0.029*	
C16	−0.1742 (3)	0.5244 (2)	−0.1468 (2)	0.0396 (8)	
H16A	−0.1724	0.5595	−0.1944	0.059*	
H16B	−0.1355	0.5552	−0.0954	0.059*	
H16C	−0.2371	0.5220	−0.1493	0.059*	
C17	0.04125 (18)	0.3342 (2)	−0.09604 (16)	0.0227 (5)	
H17	0.0200	0.3858	−0.1322	0.027*	
C18	0.12892 (18)	0.30127 (19)	−0.06317 (16)	0.0211 (5)	
H18	0.1795	0.3270	−0.0726	0.025*	
C19	0.26791 (18)	0.16265 (19)	0.27611 (15)	0.0219 (5)	
H19A	0.3352	0.1641	0.3033	0.026*	
H19B	0.2426	0.1771	0.3188	0.026*	
C20	0.23784 (18)	0.23851 (19)	0.20961 (16)	0.0208 (5)	
H20A	0.1704	0.2422	0.1862	0.025*	
H20B	0.2617	0.2994	0.2353	0.025*	
C21	0.33576 (17)	0.27120 (18)	0.13860 (15)	0.0187 (5)	
H21	0.3638	0.3194	0.1771	0.022*	
C22	0.36201 (17)	0.24818 (18)	0.06987 (15)	0.0181 (5)	
C23	0.48204 (19)	0.3653 (2)	0.07504 (19)	0.0286 (6)	
H23A	0.5271	0.3709	0.0483	0.034*	
H23B	0.5161	0.3593	0.1355	0.034*	
C24	0.4245 (2)	0.4521 (2)	0.0580 (2)	0.0361 (7)	
H24	0.4234	0.4956	0.0987	0.043*	
C25	0.2978 (2)	0.5214 (3)	−0.0437 (2)	0.0400 (8)	
H25A	0.2640	0.5246	−0.1037	0.060*	
H25B	0.3204	0.5835	−0.0226	0.060*	
H25C	0.2573	0.4989	−0.0168	0.060*	
C26	0.42143 (18)	0.2312 (2)	−0.02411 (17)	0.0246 (5)	
H26	0.4579	0.2393	−0.0562	0.030*	
C27	0.35384 (18)	0.1669 (2)	−0.03755 (16)	0.0219 (5)	
H27	0.3356	0.1217	−0.0805	0.026*	
P1	0.42153 (4)	0.71698 (5)	0.17349 (4)	0.02010 (15)	
F1	0.47941 (12)	0.70753 (14)	0.27046 (10)	0.0325 (4)	
F2	0.36482 (15)	0.72759 (15)	0.07629 (11)	0.0449 (5)	
F3	0.49897 (13)	0.66092 (13)	0.15446 (12)	0.0343 (4)	
F4	0.37056 (12)	0.62183 (12)	0.17462 (11)	0.0304 (4)	
F5	0.34493 (13)	0.77432 (14)	0.19246 (13)	0.0405 (5)	
F6	0.47373 (13)	0.81263 (12)	0.17197 (12)	0.0356 (4)	
P2	0.03848 (5)	0.82009 (5)	0.32494 (4)	0.02474 (16)	
F7	0.01914 (14)	0.92862 (13)	0.30856 (12)	0.0368 (4)	
F8	0.05638 (15)	0.71109 (14)	0.34195 (15)	0.0481 (5)	
F9	0.08112 (16)	0.81600 (16)	0.25507 (14)	0.0491 (6)	
F10	0.13629 (13)	0.84253 (17)	0.39345 (14)	0.0483 (5)	
F11	−0.00539 (13)	0.82390 (15)	0.39448 (11)	0.0378 (4)	
F12	−0.05994 (12)	0.79688 (14)	0.25731 (10)	0.0337 (4)	

### Atomic displacement parameters (Å^2^)

**Table:**

	*U*^11^	*U*^22^	*U*^33^	*U*^12^	*U*^13^	*U*^23^
Fe1	0.01229 (17)	0.01787 (19)	0.01052 (18)	0.00178 (14)	0.00231 (13)	0.00087 (14)
O1	0.0251 (10)	0.0421 (12)	0.0297 (11)	−0.0081 (9)	0.0130 (8)	−0.0144 (9)
O2	0.0355 (11)	0.0300 (11)	0.0248 (10)	0.0132 (9)	0.0143 (9)	0.0101 (8)
O3	0.0493 (14)	0.0407 (13)	0.0338 (12)	0.0089 (11)	0.0186 (11)	0.0024 (10)
N1	0.0162 (9)	0.0205 (10)	0.0129 (9)	0.0023 (8)	0.0019 (8)	0.0024 (8)
N2	0.0185 (10)	0.0237 (11)	0.0139 (10)	0.0000 (8)	0.0042 (8)	−0.0036 (8)
N3	0.0140 (9)	0.0208 (10)	0.0111 (9)	−0.0005 (8)	0.0023 (7)	0.0007 (8)
N4	0.0187 (10)	0.0247 (11)	0.0173 (10)	0.0005 (8)	0.0038 (8)	0.0004 (9)
N5	0.0172 (9)	0.0201 (10)	0.0130 (9)	0.0044 (8)	0.0045 (8)	0.0016 (8)
N6	0.0176 (9)	0.0208 (10)	0.0140 (9)	−0.0007 (8)	0.0044 (8)	−0.0003 (8)
N7	0.0170 (10)	0.0211 (10)	0.0134 (9)	0.0018 (8)	0.0044 (8)	0.0005 (8)
N8	0.0165 (10)	0.0250 (11)	0.0145 (10)	0.0051 (8)	0.0020 (8)	0.0022 (8)
N9	0.0170 (9)	0.0212 (10)	0.0137 (9)	0.0045 (8)	0.0052 (8)	0.0026 (8)
N10	0.0181 (10)	0.0239 (11)	0.0199 (10)	−0.0006 (8)	0.0064 (8)	0.0032 (9)
C1	0.0177 (11)	0.0264 (13)	0.0135 (11)	−0.0011 (10)	−0.0009 (9)	0.0013 (10)
C2	0.0213 (12)	0.0278 (13)	0.0118 (11)	−0.0021 (10)	0.0003 (9)	−0.0004 (10)
C3	0.0246 (13)	0.0250 (13)	0.0193 (12)	0.0028 (10)	0.0070 (10)	−0.0043 (10)
C4	0.0257 (13)	0.0315 (15)	0.0206 (13)	0.0000 (11)	0.0093 (11)	−0.0071 (11)
C5	0.0286 (14)	0.0434 (18)	0.0387 (17)	−0.0051 (13)	0.0177 (13)	−0.0081 (14)
C6	0.0145 (10)	0.0225 (12)	0.0137 (11)	0.0003 (9)	0.0035 (9)	0.0006 (9)
C7	0.0158 (10)	0.0210 (12)	0.0146 (11)	0.0007 (9)	0.0036 (9)	−0.0002 (9)
C8	0.0181 (11)	0.0245 (12)	0.0114 (11)	0.0039 (9)	0.0016 (9)	0.0009 (9)
C9	0.0227 (12)	0.0257 (13)	0.0115 (11)	0.0031 (10)	0.0035 (9)	0.0040 (9)
C10	0.0200 (12)	0.0318 (14)	0.0185 (12)	−0.0013 (10)	0.0072 (10)	0.0055 (11)
C11	0.0189 (11)	0.0224 (12)	0.0230 (13)	−0.0039 (10)	0.0059 (10)	0.0049 (10)
C12	0.0147 (11)	0.0236 (12)	0.0146 (11)	0.0005 (9)	0.0024 (9)	−0.0012 (9)
C13	0.0166 (11)	0.0221 (12)	0.0141 (11)	0.0026 (9)	0.0024 (9)	−0.0013 (9)
C14	0.0139 (11)	0.0291 (14)	0.0226 (13)	0.0041 (10)	0.0007 (9)	0.0045 (11)
C15	0.0210 (12)	0.0310 (14)	0.0187 (12)	0.0076 (10)	0.0060 (10)	0.0055 (11)
C16	0.0484 (19)	0.0282 (16)	0.050 (2)	0.0149 (14)	0.0279 (16)	0.0110 (14)
C17	0.0243 (13)	0.0268 (13)	0.0158 (12)	0.0050 (10)	0.0064 (10)	0.0061 (10)
C18	0.0210 (12)	0.0251 (13)	0.0164 (11)	0.0028 (10)	0.0061 (9)	0.0032 (10)
C19	0.0260 (12)	0.0268 (13)	0.0138 (11)	−0.0009 (10)	0.0085 (10)	−0.0022 (10)
C20	0.0237 (12)	0.0232 (13)	0.0181 (12)	0.0014 (10)	0.0109 (10)	−0.0030 (10)
C21	0.0180 (11)	0.0207 (12)	0.0153 (11)	0.0026 (9)	0.0039 (9)	0.0006 (9)
C22	0.0157 (11)	0.0212 (12)	0.0159 (11)	0.0022 (9)	0.0044 (9)	0.0031 (9)
C23	0.0236 (13)	0.0311 (15)	0.0293 (14)	−0.0089 (11)	0.0078 (11)	0.0017 (12)
C24	0.0379 (16)	0.0291 (15)	0.0367 (17)	−0.0025 (13)	0.0088 (14)	0.0015 (13)
C25	0.0394 (17)	0.0387 (18)	0.0374 (18)	0.0091 (14)	0.0094 (14)	−0.0064 (14)
C26	0.0224 (12)	0.0323 (15)	0.0212 (13)	0.0024 (11)	0.0104 (10)	0.0048 (11)
C27	0.0233 (12)	0.0293 (14)	0.0149 (11)	0.0026 (10)	0.0094 (10)	0.0009 (10)
P1	0.0189 (3)	0.0224 (3)	0.0168 (3)	0.0011 (2)	0.0042 (2)	0.0028 (2)
F1	0.0279 (8)	0.0445 (10)	0.0181 (8)	−0.0065 (7)	0.0004 (6)	0.0019 (7)
F2	0.0534 (12)	0.0462 (12)	0.0196 (9)	0.0020 (10)	−0.0038 (8)	0.0091 (8)
F3	0.0359 (9)	0.0329 (9)	0.0426 (10)	0.0074 (8)	0.0244 (8)	0.0055 (8)
F4	0.0274 (8)	0.0296 (9)	0.0299 (9)	−0.0083 (7)	0.0058 (7)	0.0008 (7)
F5	0.0313 (9)	0.0393 (10)	0.0544 (12)	0.0089 (8)	0.0201 (9)	−0.0018 (9)
F6	0.0390 (10)	0.0236 (8)	0.0458 (11)	−0.0041 (7)	0.0176 (8)	0.0052 (8)
P2	0.0239 (3)	0.0271 (4)	0.0206 (3)	−0.0021 (3)	0.0054 (3)	0.0010 (3)
F7	0.0443 (10)	0.0284 (9)	0.0430 (10)	−0.0032 (8)	0.0224 (9)	0.0016 (8)
F8	0.0415 (11)	0.0326 (10)	0.0684 (15)	0.0070 (8)	0.0184 (10)	0.0106 (10)
F9	0.0595 (13)	0.0526 (13)	0.0541 (13)	−0.0195 (10)	0.0431 (11)	−0.0200 (10)
F10	0.0242 (9)	0.0645 (14)	0.0450 (12)	−0.0050 (9)	0.0002 (8)	−0.0091 (10)
F11	0.0395 (10)	0.0535 (12)	0.0204 (8)	−0.0055 (9)	0.0112 (7)	0.0039 (8)
F12	0.0313 (9)	0.0427 (10)	0.0227 (8)	−0.0081 (8)	0.0051 (7)	0.0015 (7)

### Geometric parameters (Å, º)

**Table:**

Fe1—N1	1.954 (2)	C8—H8B	0.9900
Fe1—N9	1.959 (2)	C9—H9A	0.9900
Fe1—N7	1.967 (2)	C9—H9B	0.9900
Fe1—N6	1.975 (2)	C10—C11	1.532 (4)
Fe1—N5	1.978 (2)	C10—H10A	0.9900
Fe1—N3	1.989 (2)	C10—H10B	0.9900
O1—C5	1.417 (4)	C11—H11A	0.9900
O1—C4	1.421 (3)	C11—H11B	0.9900
O2—C16	1.411 (4)	C12—C13	1.442 (4)
O2—C15	1.419 (3)	C12—H12	0.9500
O3—C24	1.388 (4)	C14—C15	1.509 (4)
O3—C25	1.433 (4)	C14—H14A	0.9900
N1—C6	1.335 (3)	C14—H14B	0.9900
N1—C1	1.368 (3)	C15—H15A	0.9900
N2—C6	1.355 (3)	C15—H15B	0.9900
N2—C2	1.366 (3)	C16—H16A	0.9800
N2—C3	1.471 (3)	C16—H16B	0.9800
N3—C7	1.295 (3)	C16—H16C	0.9800
N3—C8	1.461 (3)	C17—C18	1.371 (4)
N4—C10	1.438 (3)	C17—H17	0.9500
N4—C19	1.438 (3)	C18—H18	0.9500
N4—C9	1.441 (3)	C19—C20	1.530 (4)
N5—C21	1.289 (3)	C19—H19A	0.9900
N5—C20	1.468 (3)	C19—H19B	0.9900
N6—C12	1.293 (3)	C20—H20A	0.9900
N6—C11	1.471 (3)	C20—H20B	0.9900
N7—C13	1.333 (3)	C21—C22	1.446 (3)
N7—C18	1.365 (3)	C21—H21	0.9500
N8—C13	1.351 (3)	C23—C24	1.506 (4)
N8—C17	1.374 (4)	C23—H23A	0.9900
N8—C14	1.474 (3)	C23—H23B	0.9900
N9—C22	1.328 (3)	C24—H24	0.9500
N9—C27	1.376 (3)	C25—H25A	0.9800
N10—C22	1.355 (3)	C25—H25B	0.9800
N10—C26	1.370 (4)	C25—H25C	0.9800
N10—C23	1.461 (3)	C26—C27	1.366 (4)
C1—C2	1.365 (4)	C26—H26	0.9500
C1—H1	0.9500	C27—H27	0.9500
C2—H2	0.9500	P1—F4	1.5912 (17)
C3—C4	1.512 (4)	P1—F1	1.5964 (17)
C3—H3A	0.9900	P1—F2	1.5980 (18)
C3—H3B	0.9900	P1—F5	1.5997 (19)
C4—H4A	0.9900	P1—F3	1.6021 (18)
C4—H4B	0.9900	P1—F6	1.6089 (18)
C5—H5A	0.9800	P2—F10	1.593 (2)
C5—H5B	0.9800	P2—F7	1.594 (2)
C5—H5C	0.9800	P2—F12	1.5957 (18)
C6—C7	1.437 (3)	P2—F8	1.599 (2)
C7—H7	0.9500	P2—F9	1.599 (2)
C8—C9	1.535 (3)	P2—F11	1.6077 (19)
C8—H8A	0.9900		
			
N1—Fe1—N9	90.30 (9)	H11A—C11—H11B	108.0
N1—Fe1—N7	91.96 (9)	N6—C12—C13	113.3 (2)
N9—Fe1—N7	92.27 (9)	N6—C12—H12	123.4
N1—Fe1—N6	90.75 (9)	C13—C12—H12	123.4
N9—Fe1—N6	172.91 (9)	N7—C13—N8	110.9 (2)
N7—Fe1—N6	80.69 (9)	N7—C13—C12	116.7 (2)
N1—Fe1—N5	170.70 (9)	N8—C13—C12	132.4 (2)
N9—Fe1—N5	80.58 (9)	N8—C14—C15	113.1 (2)
N7—Fe1—N5	90.29 (9)	N8—C14—H14A	109.0
N6—Fe1—N5	98.53 (9)	C15—C14—H14A	109.0
N1—Fe1—N3	80.90 (9)	N8—C14—H14B	109.0
N9—Fe1—N3	89.14 (8)	C15—C14—H14B	109.0
N7—Fe1—N3	172.73 (9)	H14A—C14—H14B	107.8
N6—Fe1—N3	97.95 (9)	O2—C15—C14	108.6 (2)
N5—Fe1—N3	96.98 (8)	O2—C15—H15A	110.0
C5—O1—C4	111.7 (2)	C14—C15—H15A	110.0
C16—O2—C15	111.3 (2)	O2—C15—H15B	110.0
C24—O3—C25	112.1 (3)	C14—C15—H15B	110.0
C6—N1—C1	106.8 (2)	H15A—C15—H15B	108.4
C6—N1—Fe1	112.71 (16)	O2—C16—H16A	109.5
C1—N1—Fe1	139.51 (18)	O2—C16—H16B	109.5
C6—N2—C2	107.2 (2)	H16A—C16—H16B	109.5
C6—N2—C3	126.1 (2)	O2—C16—H16C	109.5
C2—N2—C3	125.9 (2)	H16A—C16—H16C	109.5
C7—N3—C8	118.5 (2)	H16B—C16—H16C	109.5
C7—N3—Fe1	115.09 (16)	C18—C17—N8	106.6 (2)
C8—N3—Fe1	125.94 (16)	C18—C17—H17	126.7
C10—N4—C19	120.0 (2)	N8—C17—H17	126.7
C10—N4—C9	119.9 (2)	N7—C18—C17	109.1 (2)
C19—N4—C9	119.5 (2)	N7—C18—H18	125.4
C21—N5—C20	118.0 (2)	C17—C18—H18	125.4
C21—N5—Fe1	115.90 (17)	N4—C19—C20	114.3 (2)
C20—N5—Fe1	125.94 (17)	N4—C19—H19A	108.7
C12—N6—C11	117.7 (2)	C20—C19—H19A	108.7
C12—N6—Fe1	116.09 (18)	N4—C19—H19B	108.7
C11—N6—Fe1	126.06 (17)	C20—C19—H19B	108.7
C13—N7—C18	106.4 (2)	H19A—C19—H19B	107.6
C13—N7—Fe1	112.78 (17)	N5—C20—C19	111.8 (2)
C18—N7—Fe1	140.72 (18)	N5—C20—H20A	109.3
C13—N8—C17	107.0 (2)	C19—C20—H20A	109.3
C13—N8—C14	126.0 (2)	N5—C20—H20B	109.3
C17—N8—C14	127.0 (2)	C19—C20—H20B	109.3
C22—N9—C27	106.6 (2)	H20A—C20—H20B	107.9
C22—N9—Fe1	113.49 (17)	N5—C21—C22	113.5 (2)
C27—N9—Fe1	139.85 (19)	N5—C21—H21	123.2
C22—N10—C26	106.7 (2)	C22—C21—H21	123.2
C22—N10—C23	126.4 (2)	N9—C22—N10	110.9 (2)
C26—N10—C23	126.2 (2)	N9—C22—C21	116.2 (2)
C2—C1—N1	108.6 (2)	N10—C22—C21	133.0 (2)
C2—C1—H1	125.7	N10—C23—C24	112.0 (2)
N1—C1—H1	125.7	N10—C23—H23A	109.2
C1—C2—N2	107.2 (2)	C24—C23—H23A	109.2
C1—C2—H2	126.4	N10—C23—H23B	109.2
N2—C2—H2	126.4	C24—C23—H23B	109.2
N2—C3—C4	110.3 (2)	H23A—C23—H23B	107.9
N2—C3—H3A	109.6	O3—C24—C23	109.6 (3)
C4—C3—H3A	109.6	O3—C24—H24	125.2
N2—C3—H3B	109.6	C23—C24—H24	125.2
C4—C3—H3B	109.6	O3—C25—H25A	109.5
H3A—C3—H3B	108.1	O3—C25—H25B	109.5
O1—C4—C3	107.6 (2)	H25A—C25—H25B	109.5
O1—C4—H4A	110.2	O3—C25—H25C	109.5
C3—C4—H4A	110.2	H25A—C25—H25C	109.5
O1—C4—H4B	110.2	H25B—C25—H25C	109.5
C3—C4—H4B	110.2	C27—C26—N10	107.4 (2)
H4A—C4—H4B	108.5	C27—C26—H26	126.3
O1—C5—H5A	109.5	N10—C26—H26	126.3
O1—C5—H5B	109.5	C26—C27—N9	108.4 (2)
H5A—C5—H5B	109.5	C26—C27—H27	125.8
O1—C5—H5C	109.5	N9—C27—H27	125.8
H5A—C5—H5C	109.5	F4—P1—F1	90.33 (10)
H5B—C5—H5C	109.5	F4—P1—F2	90.56 (11)
N1—C6—N2	110.2 (2)	F1—P1—F2	179.05 (12)
N1—C6—C7	117.0 (2)	F4—P1—F5	90.73 (10)
N2—C6—C7	132.7 (2)	F1—P1—F5	90.18 (11)
N3—C7—C6	113.5 (2)	F2—P1—F5	90.13 (12)
N3—C7—H7	123.3	F4—P1—F3	90.09 (10)
C6—C7—H7	123.3	F1—P1—F3	89.82 (10)
N3—C8—C9	111.9 (2)	F2—P1—F3	89.85 (11)
N3—C8—H8A	109.2	F5—P1—F3	179.18 (11)
C9—C8—H8A	109.2	F4—P1—F6	179.42 (11)
N3—C8—H8B	109.2	F1—P1—F6	89.77 (10)
C9—C8—H8B	109.2	F2—P1—F6	89.34 (11)
H8A—C8—H8B	107.9	F5—P1—F6	89.84 (11)
N4—C9—C8	113.9 (2)	F3—P1—F6	89.34 (10)
N4—C9—H9A	108.8	F10—P2—F7	90.19 (12)
C8—C9—H9A	108.8	F10—P2—F12	179.16 (12)
N4—C9—H9B	108.8	F7—P2—F12	90.22 (11)
C8—C9—H9B	108.8	F10—P2—F8	90.28 (13)
H9A—C9—H9B	107.7	F7—P2—F8	178.96 (12)
N4—C10—C11	113.7 (2)	F12—P2—F8	89.31 (11)
N4—C10—H10A	108.8	F10—P2—F9	90.48 (13)
C11—C10—H10A	108.8	F7—P2—F9	90.41 (11)
N4—C10—H10B	108.8	F12—P2—F9	90.25 (11)
C11—C10—H10B	108.8	F8—P2—F9	90.52 (13)
H10A—C10—H10B	107.7	F10—P2—F11	90.11 (11)
N6—C11—C10	111.5 (2)	F7—P2—F11	89.62 (11)
N6—C11—H11A	109.3	F12—P2—F11	89.16 (10)
C10—C11—H11A	109.3	F8—P2—F11	89.45 (12)
N6—C11—H11B	109.3	F9—P2—F11	179.41 (12)
C10—C11—H11B	109.3		
			
C6—N1—C1—C2	−0.4 (3)	C17—N8—C13—C12	177.0 (3)
Fe1—N1—C1—C2	166.7 (2)	C14—N8—C13—C12	−4.9 (4)
N1—C1—C2—N2	0.4 (3)	N6—C12—C13—N7	−0.2 (3)
C6—N2—C2—C1	−0.2 (3)	N6—C12—C13—N8	−177.7 (3)
C3—N2—C2—C1	−170.2 (2)	C13—N8—C14—C15	133.9 (3)
C6—N2—C3—C4	−94.1 (3)	C17—N8—C14—C15	−48.3 (4)
C2—N2—C3—C4	74.0 (3)	C16—O2—C15—C14	178.2 (3)
C5—O1—C4—C3	−175.7 (2)	N8—C14—C15—O2	75.4 (3)
N2—C3—C4—O1	57.1 (3)	C13—N8—C17—C18	0.1 (3)
C1—N1—C6—N2	0.3 (3)	C14—N8—C17—C18	−178.1 (2)
Fe1—N1—C6—N2	−170.66 (16)	C13—N7—C18—C17	−0.8 (3)
C1—N1—C6—C7	177.3 (2)	Fe1—N7—C18—C17	176.0 (2)
Fe1—N1—C6—C7	6.4 (3)	N8—C17—C18—N7	0.5 (3)
C2—N2—C6—N1	−0.1 (3)	C10—N4—C19—C20	−71.8 (3)
C3—N2—C6—N1	169.9 (2)	C9—N4—C19—C20	116.5 (3)
C2—N2—C6—C7	−176.5 (3)	C21—N5—C20—C19	−107.4 (3)
C3—N2—C6—C7	−6.5 (4)	Fe1—N5—C20—C19	78.0 (3)
C8—N3—C7—C6	−179.3 (2)	N4—C19—C20—N5	−56.6 (3)
Fe1—N3—C7—C6	−6.9 (3)	C20—N5—C21—C22	−179.2 (2)
N1—C6—C7—N3	0.3 (3)	Fe1—N5—C21—C22	−4.0 (3)
N2—C6—C7—N3	176.5 (3)	C27—N9—C22—N10	0.1 (3)
C7—N3—C8—C9	−110.9 (3)	Fe1—N9—C22—N10	−177.21 (16)
Fe1—N3—C8—C9	77.5 (3)	C27—N9—C22—C21	−178.3 (2)
C10—N4—C9—C8	117.3 (3)	Fe1—N9—C22—C21	4.4 (3)
C19—N4—C9—C8	−70.9 (3)	C26—N10—C22—N9	0.5 (3)
N3—C8—C9—N4	−57.6 (3)	C23—N10—C22—N9	171.5 (2)
C19—N4—C10—C11	117.7 (3)	C26—N10—C22—C21	178.5 (3)
C9—N4—C10—C11	−70.6 (3)	C23—N10—C22—C21	−10.5 (4)
C12—N6—C11—C10	−107.0 (3)	N5—C21—C22—N9	−0.3 (3)
Fe1—N6—C11—C10	78.3 (3)	N5—C21—C22—N10	−178.2 (3)
N4—C10—C11—N6	−58.0 (3)	C22—N10—C23—C24	−62.9 (4)
C11—N6—C12—C13	179.9 (2)	C26—N10—C23—C24	106.3 (3)
Fe1—N6—C12—C13	−4.8 (3)	C25—O3—C24—C23	161.7 (3)
C18—N7—C13—N8	0.9 (3)	N10—C23—C24—O3	−56.1 (3)
Fe1—N7—C13—N8	−176.94 (16)	C22—N10—C26—C27	−0.9 (3)
C18—N7—C13—C12	−177.1 (2)	C23—N10—C26—C27	−171.9 (2)
Fe1—N7—C13—C12	5.1 (3)	N10—C26—C27—N9	1.0 (3)
C17—N8—C13—N7	−0.6 (3)	C22—N9—C27—C26	−0.7 (3)
C14—N8—C13—N7	177.5 (2)	Fe1—N9—C27—C26	175.5 (2)
